# Ten Simple Rules for the Open Development of Scientific Software

**DOI:** 10.1371/journal.pcbi.1002802

**Published:** 2012-12-06

**Authors:** Andreas Prlić, James B. Procter

**Affiliations:** 1 San Diego Supercomputer Center, University of California San Diego, La Jolla, California, United States of America; 2 School of Life Sciences Research, College of Life Sciences, University of Dundee, Dundee, Scotland, United Kingdom

Open-source software development has had significant impact, not only on society, but also on scientific research. Papers describing software published as open source are amongst the most widely cited publications (e.g., BLAST [1], [2] and Clustal-W [3]), suggesting many scientific studies may not have been possible without some kind of open software to collect observations, analyze data, or present results. It is surprising, therefore, that so few papers are accompanied by open software, given the benefits that this may bring.

Publication of the source code you write not only can increase your impact [4], but also is essential if others are to be able to reproduce your results. Reproducibility is a tenet of computational science [5], and critical for pipelines employed in data-driven biological research. Publishing the source for the software you created as well as input data and results allows others to better understand your methodology, and why it produces, or fails to produce, expected results. Public release might not always be possible, perhaps due to intellectual property policies at your or your collaborators' institutes; and it is important to make sure you know the regulations that apply to you. Open licensing models can be incredibly flexible and do not always prevent commercial software release [5].

Simply releasing the source under an open license, however, is not sufficient if you wish your code to remain useful beyond its publication [6]. The sustainability of software after publication is probably the biggest problem faced by researchers who develop it, and it is here that participating in open development from the outset can make the biggest impact. Grant-based funding is often exhausted shortly after new software is released, and without support, in-house maintenance of the software and the systems it depends on becomes a struggle. As a consequence, the software will cease to work or become unavailable for download fairly quickly [7], which may contravene archival policies stipulated by your journal or funding body. A collaborative and open project allows you to spread the resource and maintenance load to minimize these risks, and significantly contributes to the sustainability of your software.

If you have the choice, embracing an open approach to development has tremendous benefits. It allows you to build on the work of other scientists, and enables others to build on your own efforts. To make the development of open scientific software more rewarding and the experience of using software more positive, the following ten rules are intended to serve as a guide for any computational scientist.

## Rule 1: Don't Reinvent the Wheel

As in any other field, you should do some research before starting a new programming project to find out if aspects of your problem have already been solved. Many fundamental scientific algorithms and methods have already been implemented in open-source libraries, and having the source means you can easily evaluate if they will work in your situation. You can also contact online communities (see [8]) to find out about their experiences with existing approaches, and if none are appropriate, any new implementation you provide will be well received, however modest. Providing another solution to a problem, even if technologically novel, is only an accomplishment in engineering and rarely suitable for publication on its own. However, if it is useful it can benefit everyone, even if it addresses a mundane task. Furthermore, when there are no existing implementations for your platform, or they cannot cope with the size, complexity, or other specifics of your data, then new approaches may be required that lead to new science.

## Rule 2: Code Well

If you don't know them already, learn the basics of software development [9], [10]. You don't need to be the best software developer in the world, but try to be inspired by them. Study other people's code and learn by practice. Join an existing open-source project. There are plenty to choose from (most open-source repositories have a “biology” or “bioinformatics” project tag), but the “bio-*” projects hosted at the Open Bioinformatics Foundation are a good place to start [11]–[14]. Once you identify a weakness (and you will!) or something that does not work as expected, fix the issue so it works for yourself and provide a patch back to the original authors. Getting familiar with other people's code in this way is a great way to boost your experience and learn new techniques.

## Rule 3: Be Your Own User

One of the more graphic mottos in the open-source community is “eat your own dog food”. For a researcher this has two implications. If you are developing software of value to your field, it is important that you demonstrate that it can address important questions in a useful or novel way. The second implication is that your software should be useful to other developers, and is not simply a demonstration of the solution. Sadly, for some scientific software articles this is often not the case, and there are examples of software that—whilst novel—were not developed to solve a problem the scientists faced in a practical situation. Problems to do with how software is structured or functions in a variety of situations are difficult to detect during peer review. It is only later, when a researcher discovers and applies the software during their research, that these issues hinder or obstruct progress. Avoiding wasted effort of this kind is critical to researchers, who have limited time and require high levels of quality and reproducibility from scientific source code. By being “your own best user” many such problems will be detected before they become public.

## Rule 4: Be Transparent

Scientific software, like other competitive activities, is often at first developed behind closed doors instead of out in the open, and public release is then only considered around the time of publication. The first reason given for this (after any legal constraints), is the fear of getting scooped—that somebody else might use the ideas to produce competing software faster or tackle the same research problem first. In our experience, however, open development often results in just the opposite. Founding or contributing new code to open-source projects is one way for a researcher to stake a claim in a field [15]. People with similar or related research interests who discover the project will find that they have more to gain from collaborating than from competing with the original developers. The second reason given for closed development is the perhaps more serious risk that code released prematurely may lead to incorrect findings by others. However, examples regularly show [16] that even prior publication of software in a peer-reviewed journal does not preclude the presence of serious bugs. One consequence of transparent, open development is that it allows many eyes to evaluate the code and recognize and fix any issues, which reduces the likelihood of serious errors in the final product. There are public repositories such as Sourceforge or GitHub that greatly facilitate this kind of team development approach. They provide free services such as version control, Wikis, mailing lists, and bug trackers and support communication with your collaborators to share effort, document bugs, and solve problems more quickly [17]. Several models for initiating and managing open development have also been proposed and advocated by different communities, such as the Apache Way [18], [19].

## Rule 5: Be Simple

Science is hard enough already. If your software is too complex to obtain and operate or can only run on one platform, then few people will bother to try it out, and even fewer will use it successfully (particularly your reviewers!). This is doubly important for open projects, since difficult compilation or installation processes will raise a barrier against participation. Documentation helps a lot, in the form of build and installation instructions, user manuals, or even video demonstrations, but simplicity is key, since potential users will first evaluate how long it will take to install and get something out of your software against the time it will take them to find another way. Employ standard package or software installation models for as many platforms as possible. Practically all operating systems, and many languages (e.g., Perl, Ruby, and Python), have standard models for creating installable software packages, which allow you to specify any other software your code needs to run, and make it easier for you to distribute it [20]. If you don't have the time to learn how to create an installation package yourself, then get in contact with one of the many open-source packaging communities (e.g., DebianMed), and ask for help. When creating new software, try to support standard file formats and don't come up with new, custom formats. This can make your software less appealing. Spending time to create online documentation, sample data files, and test cases will give others an easy start into your codebase.

## Rule 6: Don't Be a Perfectionist

Don't wait too long with getting the first version of your source code out into the public and don't worry too much if your first prototypes still have critical features missing. If your idea is innovative, others will understand the concept. Moreover, as scientists, we are trained to constantly assess and revise our own and each others' hypotheses, and we should do the same for our software. “Release early, release often” is regarded as an open-source mantra, and attributed to Linus Torvalds by Eric Raymond [21]. It advocates the practice of releasing as soon as new work has been done, because your “customers” will quickly identify problems and new requirements, and you will be able to fix them more quickly if you avoid sitting on and polishing new code for several months before letting it into the wild. Agile development practices [22], which have become popular in the last decade, embody this iterative development process.

## Rule 7: Nurture and Grow Your Community

The biggest advantage of open development is that it allows users and developers to freely interact and form communities, and if your software is useful, your user base will grow. You can only do so much by yourself, but if you form a team (see [23]) and communicate with the people who use your tool, then new scientific and technical collaborations can arise. Reciprocity is essential, however: as a user of open source, acknowledge the tools you are using. If you are running your own open community, acknowledge the contributions of each person to your project. Make it easy for others to contribute ideas and act on feedback. Seeing that suggestions are being taken seriously and acted upon can be highly motivating and will encourage further involvement. Try to avoid changing key aspects of your code that other people's software or analysis pipelines might depend on, such as file formats, command line arguments, or application programming interfaces (APIs). If you do, discuss them online first, then document and create demonstrations of the changes, and assign a version number to the API. Even better, use Semantic Versioning (http://semver.org), which communicates both API and software version compatibility between releases. Above all, avoid confusing your users—drastic differences between each release that introduce incompatibilities will win no friends.

## Rule 8: Promote Your Project

In order to attract more attention to your project, it is important to spend time promoting it. Appearance matters, and a clean, well-organized website that will help your cause is not hard to achieve. Hosting sites such as GitHub or Google code provide standard templates for project websites, where you only need to come up with a name and logo. Branding is not rocket science, but it is about habit—once you have a name, stick with it, and use it everywhere. Create personae for your project on social networks that people can connect to, and increase your presence in online discussion forums: answer questions on ResearchGate, Linkedin, or any of the other open communities where potential users of your software might be. Whilst doing this, bear in mind that regardless of how good your project is, people are more likely to connect with your project because of what you say and your own personal profile. Finally, remember about more traditional ways of communicating your work: go to conferences where you will meet other developers and potential users of your software, and give as many presentations as you can. Keep an eye out for ad hoc developer meetups and hackathons, where open-source coders get together to work on one, or many different projects. Promotion is hard work, but through it you will grow and strengthen your community.

## Rule 9: Find Sponsors

No matter how large the community around your project and how efficiently it is developed and managed, some level of funding is essential. Scientific software can be successfully supported through grants, by writing applications to address new scientific problems through the development and use of software, or attaching development and upkeep of software as a deliverable on experimental grants. Grant writing [24] is beyond the scope of the Ten Simple Rules presented here, but it is worth mentioning that if the rules laid out here are being followed, an open development community can ensure value beyond the lifetime of an award. Open development directly addresses the section on sustainability in grant applications, but the emphasis here has to be on the community. Simply releasing code openly, without support and maintenance, will not ensure extended value; instead, you need to explain how you will actively foster your community of users and developers. Besides grants, there are also other support models for open source. Internship programs like the Google Summer of Code finance students to spend a summer working on open-source projects, and a number of projects related to science have benefited from them.

## Rule 10: Science Counts

As scientists, the software we write is primarily a means to advance our research and, ultimately, achieve our scientific goals. Whilst the development of software for the consumption of others aligns well with other processes of scientific advancement, it is the science that ultimately counts. Scientific software development fulfils an immediate need, but maintenance of code that is no longer relevant to your own research is a serious time sink, and will rarely lead to your next paper, or secure your next grant or position. Open-source development and maintenance is an intensely social process, and perhaps particularly appealing to scientists since we tend to crave interaction with others as knowledgeable about our fields as ourselves. These aspects of open source make it even more important for us as scientists to keep an eye on the big picture, and stay true to our scientific goals. However, if done right, you can publish both the science and the software for the same project, giving credit to everyone involved. Open-source communities ensure persistence of projects by allowing project leadership to be shared and passed to other members. As a scientist, this offers you the opportunity to naturally progress to new challenges with the knowledge that the software you created will remain available and benefit others.
